# Microwave-Assisted Extraction of Ricinine from *Ricinus communis* Leaves

**DOI:** 10.3390/antiox8100438

**Published:** 2019-10-01

**Authors:** Liliane Nebo, Rosa M. Varela, João B. Fernandes, Miguel Palma

**Affiliations:** 1 Laboratory of Natural Products, Department of Organic Chemistry, Federal University of São Carlos, São Carlos SP 13560-970, Brazil; lianbnb@hotmail.com (L.N.); djbf@ufscar.br (J.B.F.); 2 Department of Organic Chemistry, Faculty of Sciences, University of Cádiz, Instituto de Investigación en Biomoléculas, INBIO, 11510 Puerto Real (Cádiz), Spain; 3 Department of Analytical Chemistry, Faculty of Sciences, University of Cádiz, Instituto de Investigación Vitivinícola y Agroalimentaria, IVAGRO, 11510 Puerto Real (Cádiz), Spain; miguel.palma@uca.es

**Keywords:** microwave assisted-extraction, ricinine, *Ricinus communis*, UPLC, response surface methodology

## Abstract

The alkaloid ricinine (3-cyano-4-methoxy-N-methyl-2-pyridone) is found in different parts of the *Ricinus communis* plant and is known to possess several bioactive properties, including strong antioxidant activity. In this study, a new microwave-assisted extraction (MAE) method was developed for the recovery of ricinine from *R. communis* leaves. The extraction variables studied were extraction temperature (between 125 °C and 175 °C), microwave power (between 500 W and 1000 W), extraction time (between 5 min and 15 min), extraction solvent (between 10% and 90% of EtOAc in MeOH), and solvent-to-sample ratio (between 25:1 mL and 50:1 mL of solvent per gram of the sample). On studying the effects of extraction variables, both solvent and liquid-to-solid ratio were found to exhibit the highest effects on ricinine recovery. A fast (15 min) microwave-assisted extraction method was developed (high temperatures can be applied because the stability of ricinine is proven in the literature), allowing for the recovery of ricinine from *R. communis* leaves. The study revealed that *R. communis* leaves had almost 1.5 mg g^−1^ (dried weight) of ricinine.

## 1. Introduction

*Ricinus communis* J. (Euphorbiaceae) is a plant found in most regions of Brazil and other tropical areas [1]. Castor bean (*R. communis*) is widely used for the production of castor oil, which is used in cosmetic industries, and in the manufacturing of bio fuel. The alkaloid ricinine (3-cyano-4-methoxy-N-methyl-2-pyridone) is found in the oil, leaves, and flowers of *R. communis,* and is known to offer anticonvulsant activity [2]. Ricinine is considered a promising cognition-enhancing drug for the treatment of patients with amnesia [3]. It is responsible for the *R. communis* insecticide activity [4], specifically against leaf-cutting ants (*Atta sexdens rubropilosa*) [5] and other insects [6]. Leaf-cutting ants are one of the main herbivores in the Neotropics and are considered an huge pest to agricultural crops and forests with exotic trees, such as *Eucalyptus* ssp in Brazil. Strong antioxidant results were recently found for extracts from *R. communis* leaves, due to the high levels of ricinine [7,8].

When dealing with natural products from plants, especially those showing some kind of bioactivity or antioxidant power, the extraction methods used for the recovery of active compounds are a key step in research success [9]. Advanced extraction methodologies are recommended because of both, their higher efficiencies and lower solvent consumption. Among assisted extraction techniques, the microwave-assisted extraction technique (MAE) has been used for the recovery of antioxidants from plants [10,11] and foods [12,13]. It has been proposed as an alternative advanced method to conventional methods, specifically for the recovery of organic compounds [14]. MAE is of importance, especially if using extraction temperatures above the boiling point of solvents and high pressure to keep them in liquid phase, because higher recoveries are found using short extraction time [15]. This kind of application is possible when closed vessels are used for the extraction.

It has been demonstrated that MAE can considerably reduce both extraction time and solvent consumption, and the yields for several organic compounds can be maximized, as compared to traditional techniques [16]. MAE’s efficiency is mainly related to the selection of values for the experimental variables that affect the extraction mechanisms. The influential factors are extraction time, microwave power, temperature, solvent nature, and solvent-to-feed ratio. If there are several extraction variables that affect recovery, an experimental design can be used as a suitable tool to optimize the extraction process [17].

Accordingly, this paper presents an optimized microwave-assisted extraction (MAE) method for the extraction of ricinine from *R. communis* leaves, and the UPLC method for chromatographic determination in the extracts. This method was developed with the use of experimental design. Extraction conditions were designed to study the effect of extraction time, microwave power, temperature, solid-to-liquid ratio, and solvents on the extraction of ricinine. A previous method using dispersive solid-phase extraction and HPLC-MS was found in the literature [18]; however, it was only applied to cooking oils. In addition, a pH-zone-refining counter-current chromatography (CCC) method was published, which included the separation of ricinine from some by-products from *R. communis L.* industries, specifically from castor beans waste [19]. With regard to content in the leaves, a previous purification method, based on the high-speed counter-current technique, was developed [20]. Solid-liquid maceration for 72 h at room temperature was used to prepare the extract prior to the chromatographic separation, which is more time-consuming than the final separation step. Therefore, a fast-extraction method would be convenient in obtaining crude extracts from leaves with high levels of ricinine, therefore allowing industrial applications due to its antioxidant power and other bioactivities.

## 2. Materials and Methods

### 2.1. Reagents and Plant Material

Methanol (HPLC-grade), acetic acid (HPLC-grade), and ethyl acetate (analytical grade) were supplied by Merck (Darmstadt, Germany). Ricinine standard was purchased from Sigma-Aldrich (St. Louis, MO, USA). Water was purified using a purification system from Millipore (Milli-Q).

The leaves of *R. communis* were collected in the experimental garden of Bioscience Institute, Universidade Estadual Paulista, UNESP, Rio Claro, SP, Brazil (22°53′42.2″ S 48°29′45.6″W). The leaves of *R. communis* (approximately 1 kg in 10 different batches) were dried carefully by forced air at 40 °C to a constant weight. The dried leaves were milled with an Ultraturrax homogeneizer (IKA® T25 Digital, Germany) for 15 min prior to extraction.

### 2.2. Extraction of Ricinine

An Ethos 1600 (Milestone, Sorisole, Italy) was used for the microwave-assisted extraction experiments. Tetrafluoromethoxyl vessels with Teflon liners were used. One gram of *R. communis* leaf powder was accurately weighed. Following the experimental design, a specific volume and type of solvent was added to the extraction vessel, then the extraction was performed following specific MAE conditions. After the extraction, the vessels were cooled and the extract was filtered using a filter paper. The solution was then transferred to a volumetric flask. The extract was filtered through a 0.22 μm nylon membrane filter prior to injection on the chromatographic system.

### 2.3. Determination of Ricinine

**Figure 1:**
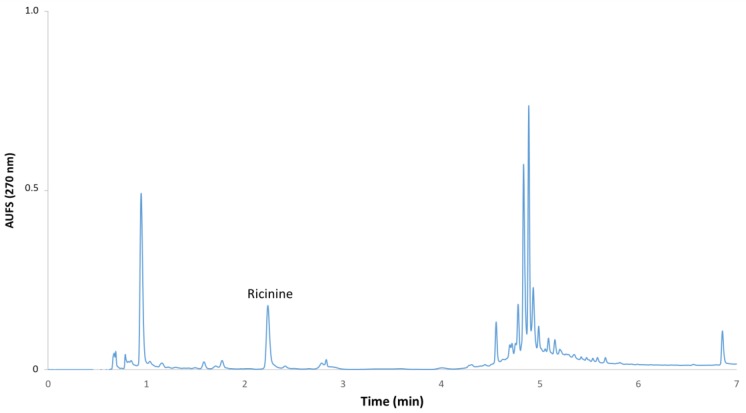
Resulting chromatogram from an extract from *R. communis* leaves.

Analyses were performed on a Waters Acquity chromatographic system coupled with a photodiode array detection method. An Acquity UPLC BEH C18 column (2.1 × 100 mm, with 1.7 μm particle size), also from Waters, was used. The column temperature was maintained at 35 °C. The binary system phases were A (2% acetic acid and 3% acetonitrile in water) and B (2% acetic acid and 85% acetonitrile in water), with a flow rate of 0.6 mL min^−1^, giving a maximum back pressure of 10400 psi, which is within the capabilities of the UPLC. The injection volume was 1.5 μL. The solvent gradient applied was as follows: 0 min, 100% A; 3–4 min, 90% A; 6.5 min, 25% A; and 6.5–7 min, 0% A up to 11 min. Finally, the column was washed with 100% B for 3 min and equilibrated with 100% A for 3 min. The identification of ricinine was carried out by comparing retention time and UV-Vis spectra of the peak obtained by the injection of ricinine standard. The resulting retention time was 2.2 min. Figure 1 shows the typical chromatogram at 270 nm from an extract from *R. communis* leaves.

Standards solutions were prepared in two different ranges from 0.5 to 10 mg L^−1^ and from 7.5 to 150 mg L^−1^, using UV detector at 270 nm Regression equations were calculated using the ALAMIN software [21]; the resulting coefficient of regression (R) was 0.999. The limits of detection (21 mg Kg^−1^) and quantification (65 mg Kg^−1^) were also calculated using the ALAMIN software.

### 2.4. Experimental Design for the Extractions

The optimization of ricinine extraction was carried out by a fractional three-level/five factor experimental design with three replicates at the central point. This kind of design was used instead of individual experiments, because it allows for the evaluation of both the individual effects and the interactive effects among the working variables. Specifically, it was used to investigate the effects of five independent variables and their interactions on the ricinine extracted from the leaves of *R. communis*. The independent variables were coded at three levels (−1, 0 and 1) and each level was selected on the basis of preliminary experiments.

The chosen levels for the variables and the fractional factorial design are provided in Table 1.

The complete experimental design consisted of 29 experimental points (Table 2), including the 3 center points (X_1_ = 150 °C, X_2_ = 750 W, X_3_ = 10 min, X_4_ = 50% EtOAc, X_5_ = 37.5 solvent to sample ratio) This technique was used to obtain the surface response by fitting the data to a polynomial model and to evaluate the effects of each factor and the interaction effects between factors.

The most general function for central composite design is represented in Equation (1).

(1) $$Y=\beta_{0}+\overset{i=5}{\underset{i=1}{\sum }}\beta_{i}x_{i}+\overset{i=5}{\underset{i=1}{\sum }}\overset{j=5}{\underset{j=1}{\sum }}\beta_{\mathrm{ij}}x_{i}x_{j}$$

where x_i_ represents the studied factors (temperature, X_1_; microwave power, X_2_; time, X_3_; solvent, X_4_; solvent/sample, X_5_); the response Y was the recovery obtained for ricinine; β_i_ (i = 1, 2,…, 5) is the parameter estimated for the factor i, β_ij_ (i = 1, 2, …, 5; j = 1, 2,…, 5) is the parameter estimated for the interaction between variables i and j, also for the quadratic effects (i = j), β_0_ is the independent factor.

### 2.5. Data Analysis

The construction and analysis of the experimental design, response surface, and desirability functions to reach the optimum conditions were obtained using Unscrambler X (CAMO) (Oslo, Norway).

## 3. Results and Discussion

### 3.1. Ricinine Stability at Different Extraction Temperatures and Microwave Powers

**Figure 2:**
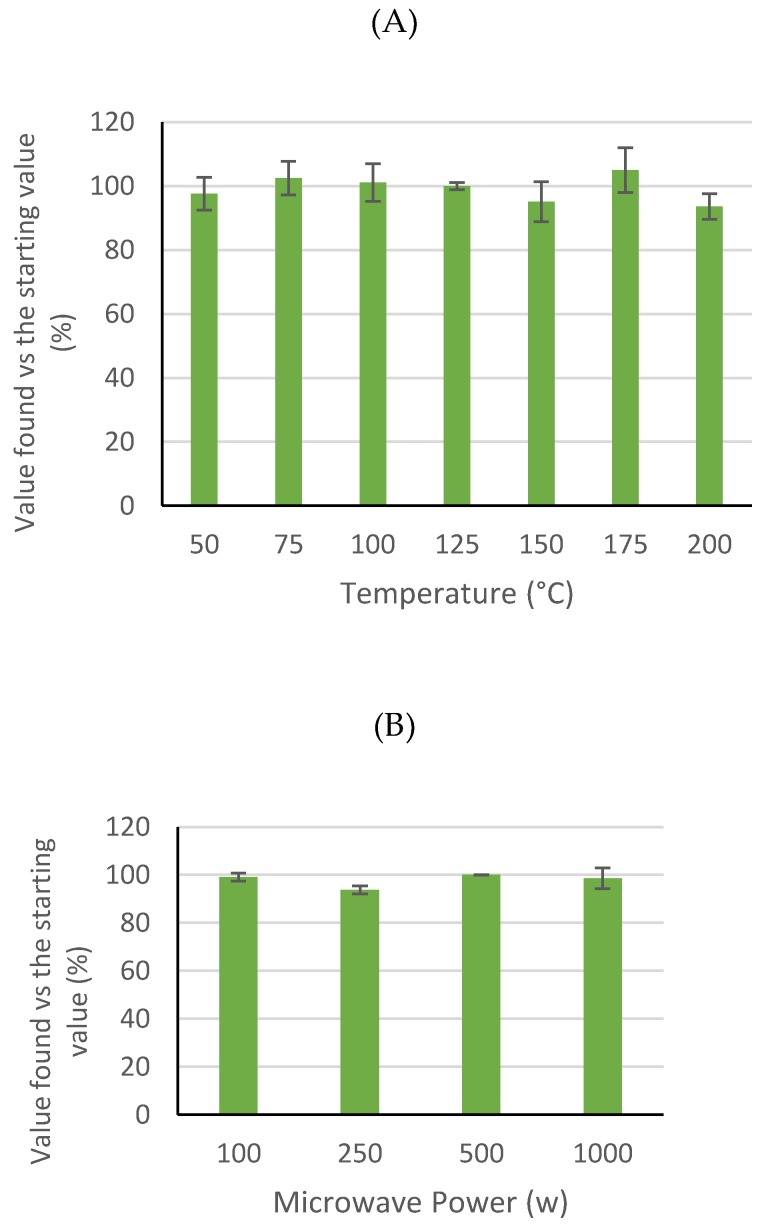
Stability of ricinine solutions (mean ± RSD; n = 3) under different microwave assisted extraction conditions: (**A**) Temperature and (**B**) Microwave Power. Values vs. the maximum level found in the solutions (%).

Prior to method development, some variables that could affect the stability of ricinine during the extraction process were investigated. Temperature was the first variable to be checked. The stability of ricinine was evaluated using methanol as a solvent at different temperatures between 50 °C and 200 °C for 10 m and microwave power of 500 watts. After applying these conditions, the remaining ricinine levels were determined in the solution (Figure 2). It can be seen that any temperature between 50 °C to 200 °C could be used for ricinine extraction without significant degradation of this compound under MAE conditions.

The effect on stability under different microwave power was also studied; Figure 2 shows the results. As can be seen, microwave power does not have significant effects on the promotion of ricinine degradation. Therefore, any value from 100 to 1000 watts could be used for microwave power for ricinine microwave-assisted extraction.

Based on the results, ricinine could be extracted using temperatures up to 200 °C and microwave power levels up to 1000 W under MAE conditions.

### 3.2. Development of the Method

An experimental design was used to optimize five factors that could affect the recovery of ricinine. The variables used in the experimental design were temperature: 125–175 °C (X_1_), microwave power: 500–1000 W (X_2_), time: 5–15 min (X_3_); solvent: 10–90% EtOAc in MeOH (X_4_) and ratio of solvent to sample: 25–50 mL/g (X_5_). Ranges for these variables were selected based on previously published papers dealing with related compounds and samples [13,17].

Three center points were included, besides the 26 regular values in the experimental design (Table 2). The yields are shown as resulting chromatographic area per gram of sample. The ricinine level measured in the leaves of *R. communis* extracts were fitted to the polynomial model following Equation (1), later calculating the predicted values.

A regression analysis was performed in order to demonstrate the empirical relationships between extraction variables in the MAE and the ricinine yield. Resulting quadratic correlation coefficient (R-Square) for the regression was 0.7152, and the Root Mean Square Error (RMSE) was 26,016, which means lower than 2% of the average value of the recovery values. The differences between the experimental values and the calculated ones are shown in Table 2. It has to be noted that the average relative error between real and predicted value was 21%; however, if values for ricinine above 50% of the highest recovery are used, average relative error is only 12%. Therefore, because the model was developed to optimize maximum recovery, it means the resulting mathematical model really fits the empirical data for the extraction conditions, showing the highest recoveries for ricinine.

Table 3 shows values for the regression coefficients for the main effects (β_i_ in Equation (1)) and the *p*-values for each term. Positive values for the regression coefficients mean the higher the value of the extraction variable, the higher the recovery of ricinine. Negative values mean the higher the value of the variable, the lower the recovery of ricinine. Interaction effects were also evaluated; however, no significant interactions were found. The significant variables with major effects were solvent (X_4_) and sample-to-solvent ratio (X_5_) because their *p*-values were lower than 0.05. Both of them show the same effect, i.e., the lower their values, the higher the recovery found for ricinine.

**Figure 3:**
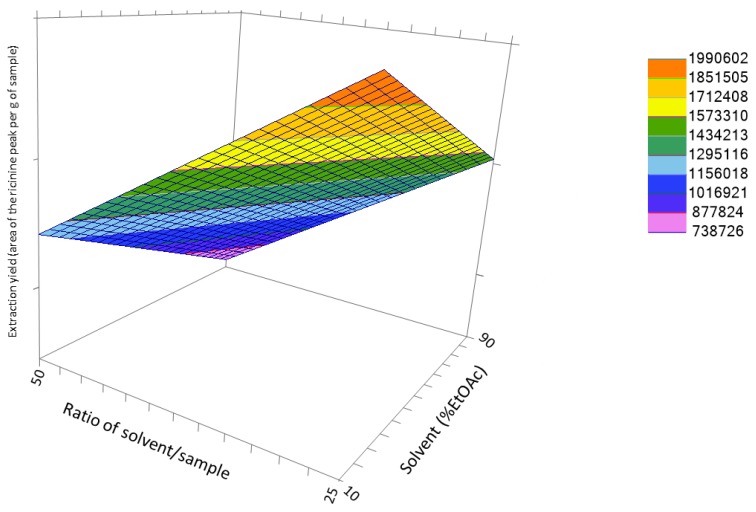
Response surface for the effects of ratio solvent to sample and solvent composition on the recovery of ricinine.

Therefore, based on the resulting model, both solvent (X_4_) and sample/solvent ratio (X_5_) should be carefully studied to reach the maximum recovery for ricinine, including values below the assayed levels. Because of extraction temperature, microwave power and extraction time showed non- significant effects; no additional experiences were run about these variables. Their regression coefficients were positive; therefore, the highest values were used for these variables for additional steps in the method development, i.e., temperature: 175 °C, power: 1000 watts, and time: 15 min. No interaction effects for these two experimental variables were found, as can be seen in Figure 3; therefore, they were checked independently.

### 3.3. Optimization of the Extraction Conditions

Because there are two extraction variables with significant effects (Table 3), both of them—solvent (X_4_) and sample/solvent ratio (X_5_)—should be evaluated in lower levels than previously assayed. Table 4 shows the resulting values for the recovery of ricinine using conditions for ratio and solvent outside the ranges used in the experimental design.

Two additional sample-to-solvent ratio values were assayed, i.e., 20 mL and 10 mL of solvent per gram of sample. It can be seen that both 20 and 10 showed significant lower recovery than the resulting recovery using 25 mL of solvent per gram of sample.

It can also be noted that lower recoveries were found for lower values of percentage of ethyl acetate in methanol, specifically 1.416 mg per gram of sample was obtained for 10% of ethyl acetate in methanol, whilst 1.263 mg and 0.525 mg per gram of sample were obtained for 5% and 0% ethyl acetate in methanol, respectively. Therefore, the final optimized extraction conditions were as it follows—temperature: 175 °C, power: 1000 watts, time: 15 min, percentage of ethyl acetate in methanol: 10%, liquid to solid ration: 25 mL g^−1^, this conditions produce near 1.5 mg of ricinine per gram of dried leaves. The value predicted by the regression model for these specific extraction conditions was 1.298 mg of ricinine per g of leaves.

### 3.4. Method Validation

Using the extraction conditions from the optimization process, the validation of the methods involving MAE followed by HPLC-DAD was accomplished. First, the precision was evaluated by studying intra-day results (repeatability), and later, inter-day results (intermediate precision). Repeatability was determined using the results from ten independent extractions on the same day. Intermediate precision was calculated using the results from five independent analyses on three consecutive days. Repeatability and intermediate precision were expressed as Coefficient of Variance (CV) of area of ricinine peak found in the extracts. The resulting CV values were less than 3% in both cases, specifically 1.44% for the repeatability (values ranged from 1.392 mg to 1.442 mg of ricinine per gram of sample) and 2.73% for the intermediate precision (values ranged from 1.385 mg to 1.491 mg of ricinine per gram of sample); therefore, the entire method, i.e., the extraction plus the separation method, have high precision. These values guarantee that the method can be applied to real samples (*R. communis*) with reliable results.

## 4. Conclusions

*R. communis* leaves showed nearly 1.5 mg g^−1^ (dried weight) of ricinine, highlighting it as an excellent source. In a previous study on *R. communis* leaves [20], a similar level was found (2.3 mg g^−1^ (dried weight); however, a much-shorter crude extract production was applied using MAE instead of solid-liquid maceration.

Microwave-assisted extraction allows for fast (15 min) recovery of ricinine from the leaves of *R. communis*. Some additional studies to scale up the method would be needed for industrial applications and in line extraction processes.

## Figures and Tables

**Table 1:** Independent variables and their levels.

Independent Variables	Codes	Variables Levels		
		−1	0	1
Temperature (°C)	X_1_	125	150	175
Power (W)	X_2_	500	750	1000
Extraction time (min)	X_3_	5	10	15
Solvent (%EtOAc)	X_4_	10	50	90
Ratio of solvent/sample	X_5_	25	37.5	50

**Table 2:** Central composite design of five variables with their observed and predicted responses.

Extraction Variable ^1^					Resulting Values for Ricinine (mg of Ricinine/g of Sample)		Relative Error (%)
X_1_	X_2_	X_3_	X_4_	X_5_	Experimental Values	Predicted Values	
175	500	5	10	50	0.483	0.496	3
175	500	5	10	25	0.870	0.901	4
175	1000	15	90	37.5	0.411	0.622	41
125	1000	5	10	50	0.390	0.545	33
150	1000	15	50	50	0.615	0.440	33
175	1000	5	10	25	0.820	0.948	15
125	1000	5	50	25	0.995	0.777	25
125	500	15	50	25	1.037	0.761	31
175	1000	15	50	25	1.003	0.845	17
175	500	15	90	37.5	0.422	0.599	35
125	750	15	90	25	0.641	0.649	1
175	1000	15	10	50	0.780	0.561	33
125	1000	10	90	37.5	0.303	0.513	52
150	1000	10	10	25	0.907	0.896	1
125	500	15	10	50	0.473	0.519	9
150	1000	5	90	25	0.683	0.685	0
150	500	15	90	50	0.415	0.336	21
175	500	10	90	25	0.776	0.706	9
175	1000	5	90	50	0.417	0.417	0
175	750	5	90	25	0.721	0.719	0
125	500	5	90	37.5	0.279	0.503	57
125	500	5	10	25	0.892	0.771	15
175	500	5	90	50	0.390	0.400	2
175	500	15	10	25	0.882	0.918	4
125	1000	15	10	37.5	0.482	0.723	40
125	750	10	90	50	0.327	0.272	18
150	750	10	50	37.5	0.582	0.615	5
150	750	10	50	37.5	0.561	0.615	9
150	750	10	50	37.5	0.585	0.614	5

^1^ X_1_ Temperature (°C), X_2_ Power (W), X_3_ Extraction time (min), X_4_ Solvent (% EtOAc), X_5_ Ratio of solvent to sample.

**Table 3:** Regression coefficients and *p*-values for the independent variables in the regression analysis.

Model Term	Estimate	*p*-Value
*b*_0_	4.0787	
*b*_1_	0.1944	0.3025
*b*_2_	0.0417	0.8330
*b*_3_	0.0360	0.8492
*b*_4_	−0.4011	0.0049
*b*_5_	−0.6843	0.0001

**Table 4:** Values of ricinine (mg. g of sample^−1^) for MAE at different ratios and solvent compositions (mean ± RSD) ^1^.

**Ratio (mL of Solvent g of Sample^−1^)**		
25	20	10
1.359 ^a^ ± 0.0079	1.173 ^b^ ± 0.0018	0.560 ^c^ ± 0.0371
**Percentage of ethyl acetate in methanol**		
10	5	0
1.416 ^a^ ± 0.0059	1.263 ^b^ ± 0.0021	0.525 ^c^ ± 0.0262

^a,b,c^ In the same line, the mean followed by different superscripts indicates significant differences (*p* > 0.05).
